# Effect of Methylene Blue on White Matter Injury after Ischemic Stroke

**DOI:** 10.1155/2021/6632411

**Published:** 2021-02-02

**Authors:** Quancheng Cheng, Xuhao Chen, Jiayi Ma, Xingyuan Jiang, Jiahui Chen, Mengqin Zhang, Yejun Wu, Weiguang Zhang, Chunhua Chen

**Affiliations:** ^1^Department of Anatomy and Embryology, School of Basic Medical Sciences, Peking University Health Science Centre, Beijing 100191, China; ^2^School of Clinical Medical Sciences, Peking University Health Science Centre, Beijing 100191, China

## Abstract

Methylene blue, the FDA-grandfathered drug was proved to be neuroprotective in ischemic stroke in rat. However, the mechanism of the protective effect was unknown. In this study, we used different animal models to investigate the effect of MB administration given within and beyond the therapeutic time window on behavioral deficits and infarct volume and related mechanism about the white matter protection. Middle cerebral artery occlusion and reperfusion (MCAO) and photothrombotic middle cerebral artery occlusion (PT-MCAO) models were used. Behavioral deficits and infarct volume were measured by foot fault test, Garcia neurological score, and TTC staining. Black gold staining and western blot were used to evaluate the brain white matter injury. We found that intraperitoneal administration of MB immediately or 24 h after the MCAO or PT-MCAO surgery reduced infarct volume, improved the neurological deficits, and reduced the white matter injury via myelin basic protein (BMP) protection. These findings suggested that MB relieved the white matter injury besides neuronal protection and has potential therapeutic effects on ischemic stroke.

## 1. Introduction

Stroke, as the second leading cause of death and disability in the world, is becoming a significant clinical concern globally. Ischemic stroke accounts for about 85% of the total incidence and principally causes brain parenchymal injury [1]. However, the collateral circulation and blood supply of the white matter are less than that of grey matter, showing extreme ischemic vulnerability of white matter. Therefore, most of the ischemic stroke caused by blockage of cerebral artery could also destroy the white matter rapidly and the volume of white matter infarction accounts for about half of the volume of cerebral infarction [2]. Currently, the treatment of ischemic stroke is primarily thrombolytic therapy with a tissue plasminogen activator [3]. However, reperfusion injury after treatment and long-term severe cognitive impairment greatly affect the prognosis and quality of patient's life. Different from neurons, myelin has stronger self-repair or self-remodel ability after injury. Therefore, targeting injured white matter might be an alternative solution for the acute ischemic stroke.

Methylene Blue (MB) was now clinically used to treat methemoglobinemia, cyanide poisoning, and ifosfamide-induced encephalopathy [4]. In recent years, researchers have found that MB could slow down the progress of some neurological disease such as Alzheimer's disease [5], Parkinson's disease [6], and traumatic brain injury [7]. Meanwhile, MB also showed neuroprotective effect for acute and chronic ischemic stroke [8, 9]. It maintains the oxygen consumption and energy supply of the brain under ischemia and hypoxic conditions by protecting mitochondria and strengthening metabolism [10]. MB could also protect neurons by regulating apoptosis, autophagy and necrosis [11]. In addition to its neuroprotective effect at the subcellular and cellular levels, MB improves the microenvironment of brain tissue, thereby increasing blood flow and sugar intake [12]. In addition to the grey matter injury, white matter is also impaired due to the oxidative stress, inflammation, and excitatory intoxication, which is a nonnegligible part of the pathogenesis of ischemic stroke. However, the role of MB on white matter injury remains unclear.

This study is aimed at investigating the functions of intraperitoneal injection of MB within and beyond therapeutic time window on infarct volume, neurological deficits, and white matter damage in rats and mice under acute ischemic stroke. Randomized, double-blinded, and solvent-controlled experiments were designed to avoid personal bias. All the mice in this study were divided randomly into different groups. The drug giver was blinded with the drug component and the neurologic examinees were blinded with the groups. The effects of MB on morphological changes were evaluated by TTC staining and behavioral tests. The effects of MB on white matter damage and related mechanism were confirmed by morphological and molecular biological experiments.

## 2. Materials and Methods

### 2.1. Animals

All procedures for this study were approved by Ethics Committee for Animal Research Studies at the Peking University Health Science Centre. Male adult Sprague-Dawley rats (280-330 g) and C57BL/6 mice (20-24 g) were acclimated for at least 3 days before surgery, with a 12-hour light/dark cycle and free to water and food.

## 3. Experimental Design

### 3.1. Experiment 1: To Observe the Effect of MB Administration within the Therapeutic Time Window on Brain Injury after MCAO

The ischemic penumbra, the brain tissue surrounding the infarcted core, is potentially salvageable if an appropriate treatment is administered within the therapeutic window [13]. In the MCAO model, the infarct core expands into more penumbra areas and reaches its maximum within 3 hours. This period is the therapeutic window, during which infarct dilation can be reduced [14]. Rats were randomly divided into Sham group (*n* = 30), MCAO group (*n* = 30), and MCAO+MB group (*n* = 30). The MCAO model was set up with the occlusion of the middle cerebral artery for 90 min using 4-0 nylon suture described as previously [15]. Based on previous report [16], we chose 10 mg/kg MB as the injection dose. When the reperfusion starts, MB solution (10 mg/kg, 0.25 g MB dissolved in 100 ml normal saline) or 4 ml/kg saline as the vehicle control was intraperitoneally injected in the MCAO+MB and MCAO groups, respectively. TTC staining, Nissl staining, black gold staining, and western blot were performed after neurobehavioral test 24 h after MCAO.

### 3.2. Experiment 2: To Observe the Effect of Multiple MB Administration beyond the Therapeutic Time Window on Brain Injury after MCAO

**Figure 1:**
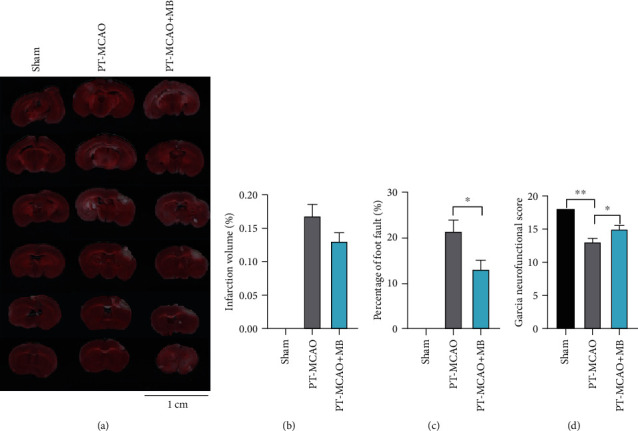
TTC staining and sensorimotor assessment in the experiment 3. (a) Representative samples of TTC stained brain sections 24 h after ischemia. No ischemic lesion was found in the Sham group. Mild cortical infarction was shown in the PT-MCAO and PT-MCAO+MB groups. The white areas represented the infarction in these sections. (b) Statistical analysis showed no significant difference between the PT-MCAO with PT-MCAO+MB groups. (c) Foot fault test showed that MB administration significantly reduced the percentage of foot fault. (d) MB administration significantly improved the postischemic sensorimotor deficit with Garcia neurofunctional scoring system. ^∗^*p* < 0.05; ^∗∗^*p* < 0.01.

**Figure 2:**
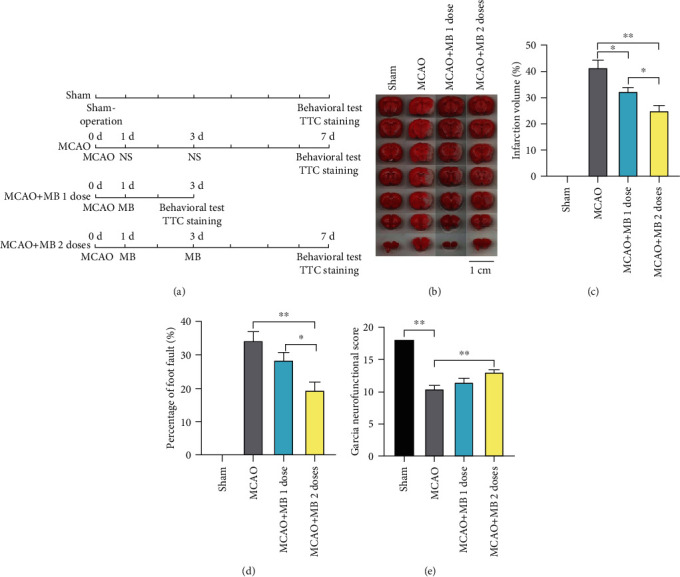
The protocol of Experiment 2, TTC staining, and sensorimotor assessment. (a) The protocol of Experiment 2. (b) Representative samples of TTC-stained brain sections. No ischemic lesion was found in the Sham group. Severe infarction was shown in MCAO mice. The white areas represented the infarction regions in these sections. MB reduced the infarction volume. MB administration with two doses is more effective than one dose. (c) Statistical analysis showed that MB administration at 24 h after MCAO with both 1 dose and 2 doses showed significantly decreased infarct volume compared with the MCAO group. (d) Foot fault test showed that rats in the MCAO group had higher foot fault rate. Meanwhile, rats in the MCAO+MB 1 dose group and the MCAO+MB 2 doses group showed significant decreased foot fault rate compared with those in the MCAO group. (e) Garcia neurofunctional score in the MCAO group, MCAO+MB 1 dose group, and MCAO+MB 2 doses group showed significant decrease compared with that in the Sham group. There was a tread of increase of the scores between the MCAO+MB 1 dose group and MCAO group, but the difference was not significant. Compared with that of the MCAO group, the score of MCAO+MB 2 doses group increased, showing a statistically significant difference. ^∗^*p* < 0.05; ^∗∗^*p* < 0.01.

The therapeutic effectiveness for human ischemic stroke decreases significantly 3 hours after stroke. Therefore, if we attempt to translate the results of basic research into clinical studies, the validation of drug efficacy beyond the therapeutic time of 3 hours is critical. As shown in Figure 1(a), the rats were divided into Sham group (*n* = 10), MCAO group (*n* = 10), MCAO+MB 1 dose group (*n* = 10), and MCAO+MB 2 doses group (*n* = 10).  Figure 2(a) showed the experimental flow chart. 10 mg/kg MB or saline was intraperitoneally injected 1 day after the operation in MCAO+MB 1 dose and MCAO groups, respectively. Rats in the MCAO+MB 2 doses group were intraperitoneally injected with 10 mg/kg MB solution on day 1 and day 3 after the operation. TTC staining was performed after neurobehavioral test on day 7 in the Sham group, MCAO group, and MCAO+MB 2 doses group. Meanwhile, TTC staining was performed on day 3 in MCAO+MB 1 dose group.

### 3.3. Experiment 3: To Verify the Effect of MB on Mice Photothrombotic Middle Cerebral Artery Occlusion (PT-MCAO) Model

Mice were randomly divided into the Sham group (*n* = 10), PT-MCAO group (*n* = 10), and PT-MCAO+MB group (*n* = 10). Mice in PT-MCAO group were injected with 10 mg/kg rose Bengal via saphenous vein, followed by stereoscopic laser irradiation and postoperative intraperitoneal injection of 4 ml/kg normal saline. Postoperative intraperitoneal injection of 10 mg/kg MB solution was performed in the PT-MCAO+MB group. TTC staining was performed after neurobehavioral test 24 h after PT-MCAO.

## 4. Establishment of Ischemic Stroke Model

### 4.1. MCAO Induced by Nylon Suture in Rats

Anaesthesia was induced and maintained with 4% and 2% isoflurane, respectively. The right common carotid artery (CCA), external carotid artery (ECA), internal carotid artery (ICA), and pterygopalatine artery (PPA) were carefully dissected in the supine position. A 4-0 suture was inserted through the incision of the ECA and was carefully pushed to a depth about 18 mm until resistance to the bifurcation was felt. After 90 min occlusion, the suture was slowly pulled out allowing for reperfusion. Rats with postoperative hemiplegia of the left forelimb were accepted to move to the next experiment. In the Sham group, the suture was inserted but immediately pulled out [17].

### 4.2. Photothrombotic Middle Cerebral Artery Occlusion in Mice

Rose Bengal, a photosensitive dye, was dissolved in sterile saline at a concentration of 20 mg/ml before the operation. The mice were fully anesthetized with isoflurane and placed in the prone position. After a middle scalp incision, the endings of the right middle cerebral artery were identified and marked with a stereomicroscope on the skull. Then, the right inguinal region of the mouse was incised to expose the great saphenous vein. Rose Bengal (10 mg/kg) was injected through this great saphenous vein. The marked artery was illuminated at 549 nm with a cold light lamp (Schot KL1500) for 1 min. Sham-operated animals underwent the same surgical procedure except the cold light illumination. The mice were housed individually after the operation. All of the procedures were performed on a thermal pad to maintain the temperature at 36.9 ± 0.5°C. Mice in the Sham group were intraperitoneally injected with the same amount of normal saline 5 minutes before illumination, and the rest steps were the same as those in the model group.

## 5. The Sensorimotor Assessment

### 5.1. Foot Fault Test

Behavioral tests were performed before sample harvest. The rats were placed on a grid floor with the size of 45 × 27 cm (with grid opening of 3 × 3 cm^2^). And the rats were recorded with a camera for 5 minutes or 50 steps. The limb falling through the grid was regarded as foot fault. The number of foot fault of the left forelimb was counted and compared with the total steps.

### 5.2. Garcia Neurofunctional Score

The scores were calculated from the following six tests: spontaneous activity, symmetry in the movement of all four limbs, forepaw outstretching, climbing, body proprioception, and response to the whisker stimulation. The lower score means the more serious of the neurological deficits.

### 5.3. 3,5-Triphenyltetrazolium Chloride (TTC) Staining

The brain was removed after sacrifice. Coronal sections of the brain (2 mm/slice, rats; 1 mm/slice, mice) were cut and immersed in 2% solution of TTC for 30 min at 37°C. After fixation with 4% paraformaldehyde, the slices were photographed and the infarction rate was calculated as follows: white area of infarcted hemisphere/red area of contralateral hemisphere × 100%.

## 6. Tissue Section Preparation

The brain was fixed with 4% paraformaldehyde, dehydrated in 30% sucrose, and embedded in OCT at low temperature. The brains were cut into slices (10 *μ*m thick), and sections were stored at -20°C for the black-gold and Nissl staining.

## 7. Black-Gold Staining

After rinsing in distilled water, the slices were dipped in 0.3% black-gold solution for 30 min in an oven (60°C). The sections were then rinsed in distilled water, incubated in 1% sodium thiosulfate for 3 min, and counterstained with Nissl solution.

## 8. Nissl Staining

The morphological changes of neurons in the hippocampus of ischemic hemisphere were observed by Nissl staining. The slices were dipped in Nissl solution for 30 min in a 37°C incubator and dehydrated through ascending series of ethanol (dipped in 70%, 80%, and 90% and twice in 100% ethanol, for 30 s each).

## 9. Western Blot

Based on a previous report [18], the brain tissues in ischemic penumbra were homogenized and lysed in ice-cold RIPA buffer for 15 min. The lysate was centrifuged at 12,000 g for 10 min at 4°C. 30 *μ*L protein was separated by SDS-PAGE analysis gel. Then the separated protein was migrated to PVDF membranes and was blocked in 5% skim milk dissolved in Tris-buffered saline-Tween-20 (TBST) for 1 h at room temperature. The membranes were incubated with antibodies of rabbit anti-Bim (ab32158), rabbit anti-MBP (ab40390), mouse anti-CNPase (ab6319), and mouse anti-GAPDH (ab8245) overnight at 4°C. Then, the membrane was incubated with horseradish peroxidase-conjugated anti-rabbit or anti-mouse IgG antibody for 1 h at room temperature. The membrane was visualized using a super-enhanced chemiluminescence reagent (ECL).

## 10. Statistical Analysis

Data were expressed as the mean ± SEM. SPSS 22 software was used for data analysis. GraphPad Prism 7 was used for statistical image processing. Multiple comparisons were statistically analysed with one-way analysis of variance (ANOVA) followed by Tukey multiple comparison post hoc analysis. *p* < 0.05 was considered significant.

## 11. Results

### 11.1. MB Administration within the Therapeutic Time Window Reduced the Infarct Volume and Improved the Sensorimotor Deficit after MCAO

**Figure 3:**
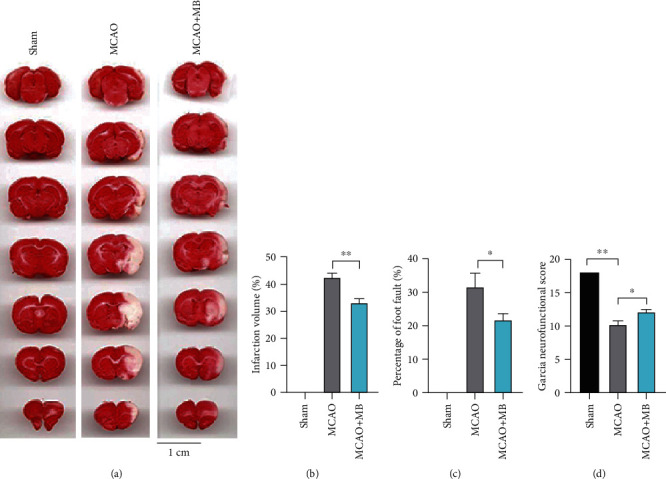
TTC staining, infarction ratio of rat brain, percentage of foot fault, and Garcia neurofunctional scores in Experiment 1. (a) Representative samples of TTC-stained brain section at 24 h after MCAO. No ischemic lesion was found in the Sham group. Severe infarction was shown in MCAO mice. The white areas represented the infarct regions in these sections. MB reduced the infarction volume. (b) Statistical analysis of the infarction ratio, which was calculated as follows: the infarcted volume of the ipsilateral hemisphere divided by the total contralateral hemisphere. The statistical analysis showed that MB significantly reduced the infarction volume. (c) Percentage of foot fault. MB significantly reduced the percentage of foot fault. (d) Grades of 3-18 were used for Garcia neurofunctional scores. MB significantly improved the postischemic sensorimotor deficit. ^∗^*p* < 0.05; ^∗∗^*p* < 0.01.

Cerebral infarction of brain slices in different groups was observed with TTC staining as shown in Figure 3(a). Using statistical analysis, we found that the infarct volume of MCAO+MB group was reduced significantly compared with that of the MCAO group, which is also significantly higher compared with that of the Sham group (Figure 3(b), *p* < 0.05). The foot fault test and Garcia neurofunctional score showed a significant difference between the MCAO and Sham groups and between the MCAO and MCAO+MB groups (Figures 3(c) and 3(d), *p* < 0.05). Briefly, 24 h after MCAO, sensorimotor deficits were significantly improved after MB intervention immediately.

### 11.2. MB Administration within the Therapeutic Time Window Protected the Damaged Myelin besides Neurons of the Cortex and Basal Ganglia

**Figure 4:**
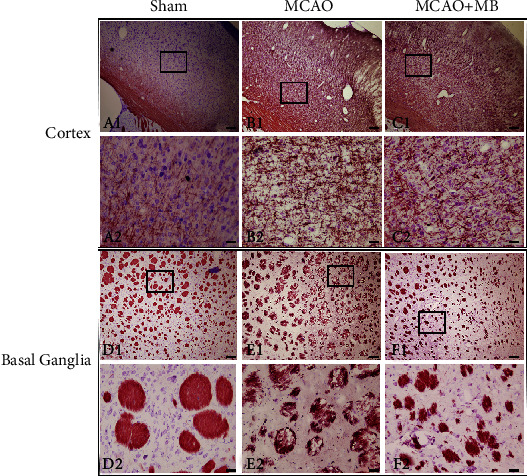
Black-gold staining of cortex and basal ganglia 24 h after ischemia-reperfusion. Black-gold staining of cortical myelin sheath and neurons in the Sham, MCAO, and MCAO+MB groups is shown in images A, B, and C, respectively. A2–C2 were the higher magnifications of the area outlined in A1–C1. Myelin sheath and neurons in basal ganglia in the Sham, MCAO, and MCAO+MB groups are shown in images D, E, and F, respectively. D2–F2 were the higher magnifications of the area outlined in D1–F1. Intact neurons and myelin sheath were observed, and Nissl body could clearly be seen in the Sham group. The MCAO injury destroyed the integrity, accompanied by reduced Nissl body and disordered myelin arrangement. However, the Nissl body in the MCAO+MB group was slightly increased, and the myelin sheath is relatively intact. Scale bar: 250 *μ*m.

Black-gold staining was used to observe the histopathological changes of myelin of cerebral cortex and basal ganglia after stroke. Intact neurons and myelin sheath were arranged in order, and Nissl body could clearly be seen in the Sham group in the cortex and basal ganglia (cortex: Figure 4 A1 and A2; basal ganglia: Figure 4 D1 and D2). Cortical integrity was destroyed with significant neuronal shrinkage, accompanied by reduced Nissl body and disordered myelin arrangement and partial deletion in the MCAO group (cortex: Figure 4 B1 and B2; basal ganglia: Figure 4 E1 and E2). However, the Nissl body in the MCAO+MB group was slightly increased and the myelin sheath is relatively intact (cortex: Figure 4 C1 and C2; basal ganglia: Figure 4 F1 and F2).

### 11.3. MB Administration within the Therapeutic Time Window Alleviates Hippocampal Neuronal Injury

**Figure 5:**
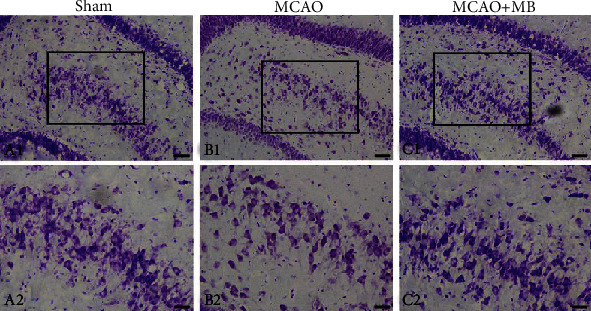
Nissl staining of hippocampal 24 h after ischemia-reperfusion. The neurons in the Sham group were well organized with obvious Nissl body (A). Neurons in the MCAO group showed disordered arrangement, with less Nissl body (B). The number of neurons in the MCAO+MB group increased and the arrangement was more compact (C). A2–C2 were the higher magnifications of the area outlined in A1–C1. Scale bar: 250 *μ*m.

The damage of pyramidal cells in hippocampal dentate gyrus region after stroke was evaluated by Nissl staining. The cells of Sham group are well organized, orderly, and regular in morphology, rich in Nissl bodies, which were in the shape of dark blue particles or plaques (Figure 5 A1 and A2). The MCAO group showed increased cell gaps, disordered arrangement, irregular morphology, Nissl bodies disintegrated or lost, and scattered distribution (Figure 5 B1 and B2). The number of cell layers in the MCAO+MB group increased, and the arrangement was more compact. The pathological morphology was improved, and the number of Nissl body was increased (Figure 5 C1 and C2).

### 11.4. MB Administration Alleviated White Matter Injury by Inhibiting Apoptotic Pathway

**Figure 6:**
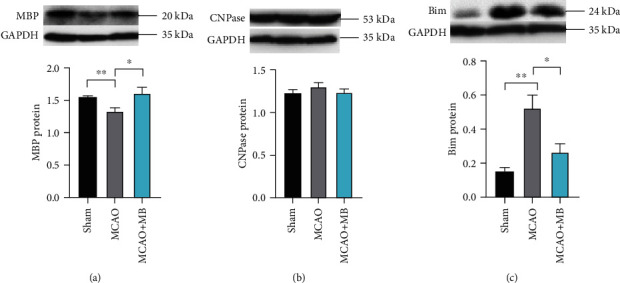
The representative images and quantification analysis of western blot of MBP, CNPase, and Bim protein. Compared with those in the Sham group, the expression of MBP and Bim in the MCAO group was significantly decreased and increased, respectively. MB administration reversed the changes significantly (a, c). There was no significant change of CNPase expression (b). ^∗^*p* < 0.05; ^∗∗^*p* < 0.01.

The protein level of myelin basic protein (MBP) and 2′,3′-cyclic-nucleotide3′-phosphodiesterase (CNPase) as the white matter marker was detected by western blot. Compared with the Sham group, MBP expression in the MCAO group was significantly reduced, and the difference was statistically significant. After MB administration, MBP expression was significantly increased compared with MCAO group (Figure 6(a), *p* < 0.05). However, there was no difference of CNPase expression among the three groups (Figure 6(b)). The expression of Bim, which is one apoptotic-related protein in the MCAO group, was significantly increased compared with that of the Sham group. After MB administration, Bim expression was significantly reduced compared to that of the MCAO group (Figure 6(c), *p* < 0.05). The results showed that MB administration within the therapeutic time window not only alleviated white matter injury but also affected the regulation of apoptosis. And the regulation of apoptosis might contribute to the protection of ischemic tissue.

### 11.5. MB Administration beyond the Therapeutic Time Window Alleviated Brain Injury

To further clarify the therapeutic effect of MB, cerebral infarction and behavioral changes in rats were evaluated 3 days after MCAO (1 dose) and 7 days after MCAO (2 doses) (Figure 2(a)). Cerebral infarction was evaluated by TTC staining of brain slices. The infarction volume of the MCAO group was significantly increased compared with that of the Sham group. MB administration at 24 h after MCAO (MCAO+MB 1 dose group) and at 24 h and 48 h (MCAO+MB 2 doses group) showed significantly decreased infarct volume compared with that of the MCAO group (Figures 2(b) and 2(c), *p* < 0.05). A foot fault test showed that rats in the MCAO group had higher foot fault rate. Meanwhile, rats in the MCAO+MB 1 dose group and the MCAO+MB 2 doses group showed significant decreased foot fault rate compared with those in the MCAO group (Figure 2(d), *p* < 0.05). Garcia neurofunctional score in the MCAO group, MCAO+MB 1 dose group, and MCAO+MB 2 doses group showed significant decrease compared with that in the Sham group. There was a tread of increase of the scores between the MCAO+MB 1 dose group and MCAO group, but the difference was not significant (Figure 2(e), *p* > 0.05). Compared to that of the MCAO group, the score of MCAO+MB 2 doses group increased, showing a statistically significant difference (Figure 2(e), *p* < 0.05).

### 11.6. MB Administration Alleviated Brain Injury in PT-MCAO Model in Mice

The mouse PT-MCAO model was used to evaluate the effect of MB on neurobehavior and cerebral infarction to further verify the neuroprotective effect of MB. TTC staining showed obvious cerebral infarct lesions in the MCAO and the MCAO+MB group (Figure 1(a)). Although there was no statistical difference between the MCAO and MCAO+MB groups, the infarct volume in the MB group was reduced to a certain extent (Figure 1(b), *p* > 0.05). The foot fault test and Garcia neurofunctional score showed significant difference between the MCAO group and Sham group. Meanwhile, there was also significant difference between the MCAO+MB and the MCAO group (Figures 1(c) and 1(d), *p* < 0.05), indicating that there were obvious behavioral deficits after MCAO, and MB intervention significantly improved the behavioral deficits.

## 12. Discussion

This study mainly found that MB administration within and beyond therapeutic time window can ameliorate the sensorimotor impairments caused by MCAO and reduce the cerebral infarction by alleviating white matter damage. We also found that MB interferes with the repairing process of injured myelin by regulating apoptotic pathways.

The white matter is mainly composed of myelin sheathed neuronal axons and oligodendrocytes [19]. White matter is just as vulnerable to ischemic damage as grey matter. However, damage to white matter has been largely ignored. In ischemic stroke, oligodendrocyte apoptosis, loss of myelin sheath, and nerve fibre rupture lead to white matter injury and vacuolated lesions [2]. Different from neurons, myelin has a strong self-repair and remodel capacity after injury. Until now, no appropriate drug intervention was developed which caused failure of myelin repair of white matter injury. In our experiment, we observed white matter was significantly damaged after MCAO, with sparse myelin sheath, disordered arrangement, and partial deletion, which was basically consistent with the previous reports [20, 21].

MB could increase oxygen consumption and energy supply to the brain by protecting mitochondria and strengthening metabolism [6]. Neuroprotective effect of the penumbra area of ischemic hemisphere was regulated by regulating autophagy and apoptosis [8] and anti-inflammation [22]. In this study, we found that MB administration could significantly reduce neurobehavioral deficits and infarct volume, thus verifying its neuroprotective effect. We also found that MB significantly reversed neuronal damage in the hippocampus by Nissl staining. Meanwhile, MB significantly reduced the myelin damage in the cortex and basal ganglia which suggested that the effect of MB on brain injury is largely achieved by reducing the white matter damage.

MBP is the main protein of myelin in the central nervous system (CNS), which is a white fatty substance that forms a medullary sheath around the axis cylinder of some nerve fibres [23]. In this study, MCAO reduced MBP expression of the ischemic hemisphere, which proves the existence of white matter lesion at the molecular level. Previous studies focused more on the recovery of grey matter than white matter injury. Studies of MB also focused on the neuroprotective effect of grey matter caused by neurodegenerative disease or ischemic injury [24–26]. This study firstly confirmed that MB could significantly alleviate the MBP reduction of myelin caused by ischemic injury. CNPase, which is abundant in myelin, plays a key role in the axonal formation of oligodendrocytes. Ramos Cejudo et al. confirmed the downregulation of CNPase in the white matter in the endothelin-1-induced cerebral ischemia model [27]. However, in this study, there was no significant change in CNPase expression, which was inconsistent with the changes of MBP expression. We hypothesized that the short ischemia time only caused the destruction of the existing myelin sheath, but the myelin formation process of oligodendrocytes was not further destroyed. CNPase downregulation in white matter confirmed by Ramos Cejudo et al. is found in chronic cerebral ischemia rather than acute cerebral ischemia model in our study. We preliminarily detected the changes of the expression of Bim, a key protein in the apoptotic pathway, in the ischemic penumbra and found that MB could significantly reverse the elevation of Bim induced by MCAO. This suggests that MB might affect the myelin repair by interfering the apoptotic pathway.

Timely MB administration could significantly alleviate brain injury after ischemia. However, most of patients of ischemic stroke could not receive timely treatment within 3-6 hours in the clinical situation. Therefore, it is of great significance to further study the effect of MB in the extended therapeutic time window. In this study, MB administration at 24 h after modelling reduced cerebral infarction and improved neurobehavioral deficits, which indicated that MB treatment had considerable effects with broadened therapeutic time window. This observation was consistent with the previous study about oral administration of MB (4 mg/kg) for 21 days [9]. We also found that MB had a dose-dependent effect on the treatment of brain injury. The results showed that the effect could be better if the drug was used repeatedly beyond the optimal therapeutic time window. However, in this study, intraperitoneal injection was used, which was more efficient than oral administration. Moreover, the dosage was 10 mg/kg, so the therapeutic effect was significant in the short time. Since MB had hormesis effect with high dose [28], long-term and higher doses were not recommended.

In this study, two MCAO models were used to confirm the neuroprotective effect of MB in ischemic stroke from the different perspective. Failure to achieve reperfusion in PT-MCAO model simulated the clinical practice when patients missed the opportunity of thrombolytic therapy. The brain injury in PT-MCAO is mild with low behavioral deficits and small cerebral infarction volume. But MB administration still showed definite therapeutic effect with improved sensorimotor deficits which suggested that MB has great potential as a therapeutic agent for ischemic stroke. Two MCAO models were used to further verify the therapeutic effect of MB and increase the reliability in the practice.

Although MB is known to be neuroprotective, this study also identified its important role in myelin repair. However, the specific target of MB and the mechanism of regulation of white matter repair need further exploration to support. In addition to the intervention of apoptosis-related pathways, other signalling pathway might participate in the process of myelin repair.

## 13. Conclusions

To summarize, MB administration could alleviate the ischemic brain injury by protecting the white matter besides neuroprotection and has potential application value in clinical treatment of ischemic stroke.

## Data Availability

The raw data supporting the findings of this study are available from the corresponding author on reasonable request.
